# Surveillance of high-risk early postsurgical patients for real-time detection of complications using wireless monitoring (SHEPHERD study): results of a randomized multicenter stepped wedge cluster trial

**DOI:** 10.3389/fmed.2023.1295499

**Published:** 2024-01-05

**Authors:** Linda M. Posthuma, Martine J. M. Breteler, Philipp B. Lirk, Els J. Nieveen van Dijkum, Maarten J. Visscher, Jennifer S. Breel, Carin A. G. L. Wensing, Jimmy Schenk, Lyan B. Vlaskamp, Mathilde C. van Rossum, Jelle P. Ruurda, Marcel G. W. Dijkgraaf, Markus W. Hollmann, Cor J. Kalkman, Benedikt Preckel

**Affiliations:** ^1^Department of Anesthesiologie, Amsterdam University Medical Center, Location University of Amsterdam, Amsterdam, Netherlands; ^2^Department of Anesthesiologie, University Medical Center, Utrecht, Netherlands; ^3^Department of Anesthesiologie, Perioperative and Pain Medicine, Brigham and Women’s Hospital, Harvard Medical School, Boston, MA, United States; ^4^Department of Surgery, Amsterdam University Medical Center, Location University of Amsterdam, Cancer Center Amsterdam, Amsterdam, Netherlands; ^5^Department of Epidemiology and Data Science, Amsterdam University Medical Center, Location University of Amsterdam, Amsterdam, Netherlands; ^6^Amsterdam Public Health, Quality of Care, Amsterdam, Netherlands; ^7^Biomedical Signals and Systems, University of Twente, Enschede, Netherlands; ^8^Department of Gastro-Intestinal and Oncologic Surgery, University Medical Center Utrecht, Utrecht, Netherlands; ^9^Department of Epidemiology and Data Science, Amsterdam University Medical Center, Location AMC, Amsterdam, Netherlands; ^10^Amsterdam Public Health, Methodology, Amsterdam, Netherlands; ^11^Amsterdam Cardiovascular Science, Diabetes and Metabolism, Amsterdam, Netherlands

**Keywords:** patient monitoring, early warning score, critical care, quality control, vital sign, postoperative complication

## Abstract

**Background:**

Vital signs measurements on the ward are performed intermittently. This could lead to failure to rapidly detect patients with deteriorating vital signs and worsens long-term outcome. The aim of this study was to test the hypothesis that continuous wireless monitoring of vital signs on the postsurgical ward improves patient outcome.

**Methods:**

In this prospective, multicenter, stepped-wedge cluster randomized study, patients in the control group received standard monitoring. The intervention group received continuous wireless monitoring of heart rate, respiratory rate and temperature on top of standard care. Automated alerts indicating vital signs deviation from baseline were sent to ward nurses, triggering the calculation of a full early warning score followed. The primary outcome was the occurrence of new disability three months after surgery.

**Results:**

The study was terminated early (at 57% inclusion) due to COVID-19 restrictions. Therefore, only descriptive statistics are presented. A total of 747 patients were enrolled in this study and eligible for statistical analyses, 517 patients in the control group and 230 patients in the intervention group, the latter only from one hospital. New disability at three months after surgery occurred in 43.7% in the control group and in 39.1% in the intervention group (absolute difference 4.6%).

**Conclusion:**

This is the largest randomized controlled trial investigating continuous wireless monitoring in postoperative patients. While patients in the intervention group seemed to experience less (new) disability than patients in the control group, results remain inconclusive with regard to postoperative patient outcome due to premature study termination.

**Clinical trial registration:**

ClinicalTrials.gov, ID: NCT02957825.

## 1 Introduction

Perioperative complications are associated with prolonged morbidity, new disability, and mortality. Patient outcome highly depends on two factors: early detection of complications and their timely and effective treatment (1, 2).

Rapid Response Teams (RRT) have been introduced to improve timely and effective treatment of deteriorating patients. In current clinical practice, detection of complications on general patient wards still relies on intermittent (every 8–12 h) assessment of vital signs and patient condition by nursing staff (3). As a result of this low monitoring frequency, deterioration of patients may often go unnoticed for prolonged periods of time (4). For instance, in the study of Sun et al. (5), continuously measured peripheral oxygen saturation in ward patients showed that one third of patients had an oxygen saturation below 90% for more than 1 h. Of these patients, only in 5% of the flow sheets, hypoxemia was recorded. In the study of Turan et al. (6), continuously measured blood pressure showed that a quarter of patients experienced a period of hypotension for at least 30 min. Routine monitoring missed half of these patients. Using continuous monitoring in patients undergoing major abdominal cancer surgery it was shown that very low SpO_2_ and tachycardia in postoperative patients are common and underdiagnosed by normal spot check monitoring (e.g., event rates for tachycardia determined by continuous monitoring 60% vs. 6% with standard monitoring) (7). In many cases of patient deterioration, measurable changes in vital signs could have identified those patients at risk for developing a certain complication already hours earlier (8–10). Delay in diagnosis can result in otherwise preventable admissions to the intensive care unit (ICU) (11). In addition, timely detection of severe sepsis may result in a shorter average length of hospital stay and early initiation of adequate treatment (12). Wireless and wearable devices that continuously track patients’ vital signs could improve the timeliness of recognizing patient deterioration, thereby theoretically minimizing *failure to detect* patient deterioration (13). Small randomized controlled trials or before-after studies in postoperative patients showed that continuous vital signs monitoring is associated with a reduced need of ICU transfers or reduction in length of hospital stay (11, 14–17). However, whether there is an improvement in long-term outcome has not yet been convincingly proven, and prospective multicenter randomized trials are lacking (12).

The aim of this study was therefore to test the working hypothesis that in general ward patients undergoing intermediate or high-risk surgery (18, 19), continuous wireless monitoring during the first five post-operative days in addition to routine monitoring improves post-operative outcome, as measured by reduced new-disability three months after surgery.

## 2 Materials and methods

### 2.1 Study design

The SHEPHERD study was an interventional, multicenter, prospective, controlled trial following a cluster stepped-wedge design in patients on post-surgical wards, at two academic centers in the Netherlands (Amsterdam University Medical Center, Location University of Amsterdam, and University Medical Center Utrecht).

The medical ethics committee of Amsterdam University Medical Center approved the study protocol (project ID NL59154.018.16). The sponsor of the study was Amsterdam University Medical Center. The study was registered at ClinicalTrials.gov (ID: NCT02957825; 02-02-2018). All participating patients gave written informed consent for study participation. The study was monitored by the Clinical Research Unit of Amsterdam UMC. This study adhered to the principles of the Declaration of Helsinki (Fortaleza) and Good Clinical Practice (GCP). The CONSORT guidelines were used in writing of this manuscript.

### 2.2 Eligibility criteria; in-and exclusion criteria

Patients undergoing non-cardiac intermediate or high-risk surgery (18, 19) were included if they were between 18 and 99 years old and had an American Society of Anesthesiologists (ASA) physical status classification I to IV. Patients were excluded if written informed consent was not obtained.

### 2.3 Interventions and study outline

Recruitment of patients was performed by an independent investigator during the preoperative evaluation period.

*The control group* received standard monitoring according to the hospital protocol consisting of intermittent visits of nurses and physicians. Modified Early Warning Scores (MEWS) (20) were calculated by hand every 8 h in both hospitals, or more frequently if indicated according to standard operating procedures (Supplementary Appendix 1).

*The intervention group* received standard monitoring plus continuous wireless monitoring (see below). Continuous wireless monitoring started after discharge from the Post Anesthesia Care Unit (PACU) and was continued for five days or until discharge. If the respective vital signs deviated from the pre-set alarm limits for more than 14 min, an audio notification was sent to the nurses’ handheld communication device. Pre-set alarm limits for HR were < 40 and > 120 beats per minute, for RR below <8 or above >24 breaths per minute, and for temperature > 39.0°C. System disconnections were displayed on the mobile communication device of the attending nurses.

### 2.4 Experimental protocol

**Figure 1:**
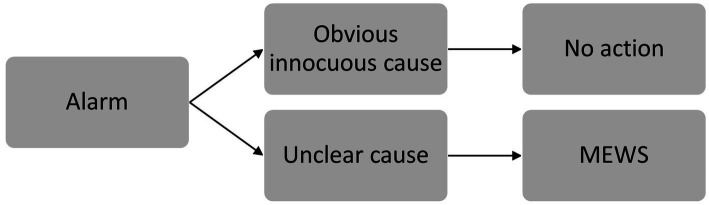
Steps taken after alarm triggering. If there were obvious but innocuous causes for the alarm (e.g., tachycardia during mobilization or physiotherapy), no action was required. In all other cases, the nurse was requested to calculate the MEWS.

When the wireless system generated a notification, the nurse was requested to evaluate the alarm and/or see and inspect the patient. If there were obvious but innocuous causes for the alarm (e.g., tachycardia during mobilization or physiotherapy), no intervention was required (Figure 1). If vital signs were deviated over a longer time period, but there was no postoperative complication after evaluation, nurses were allowed to optimize alarm settings. In all other cases, the nurse was requested to measure vital sings and calculate the MEWS. Actions taken in case of an abnormal MEWS are established per hospital (escalation) protocol (Supplementary Appendix 1) (21).

*Continuous wireless monitoring* was performed using the SensiumVitals^®^ (Sensium Healthcare, Oxford, United Kingdom) adhesive patch sensor. This system measures heart rate, respiratory rate, and axillary temperature every 2 min. A detailed description of how the system works was described in a previous study (22). The continuous wireless monitoring of SensiumVitals^®^ was selected for this study because it had an CE-certificate when study protocol was written. Also, this system measures respiratory rate and heart rate, vital signs that are considered to be the best predictors of patient deterioration (23). Furthermore, the accuracy of vital sign measurement by the system was validated before the study. For both, respiratory rate and heart rate, a mean difference of less than one breath or one beat per minute, respectively, was shown (22). Previous analysis showed that the SensiumVitals system is able to deliver at least 67% valid measurements per day (24); the system was previously tested clinically (25) and does not restrict patient mobilization.

According to the stepped-wedge design (see below), all patients subjected longer than 24 h to the respective wards (even those not included into study analysis) received a vital signs patch sensor during the intervention period. This means that when the intervention period started on a specific ward, remote monitoring (on top of standard monitoring) became standard of care for all admitted patients, regardless of study participation.

### 2.5 Outcome measurement

Postoperative complications that result in new disability as perceived by patients could determine what constitutes to medical success or failure (26). Early recognition of aberrant vital signs of patient by wireless wearable vital signs monitoring might provide an opportunity to prevent further worsening of a patient’s condition, which could prevent new disability. Shulman et al. (26) proposed “new disability” as a truly patient-centered outcome that can be used as a valid endpoint or primary outcome in perioperative outcomes research. This primary outcome is a combination of survival and a patient-reported assessment of disability measured by a validated questionnaire, the World Health Organization Disability Assessment Schedule (WHODAS) 2.0 (12-item version) (27). The WHODAS provides a single outcome of the burden of disability, caused by different postoperative complications or worsening of pre-existent morbidity. A change in the WHODAS score of 5% or more after surgery is considered a clinically important change in disability (28). Results of this questionnaire measured three months after surgery, and compared to pre-operative status, were used for the primary outcome of the present study. Disabilities were registered by the WHODAS before and after surgery, enabling distinguishing between pre-existent and new disabilities. Patients who died were classified as having new disability.

Secondary outcome measurements included mean disability at one month after surgery (WHODAS 2.0), percentage of patients with at least moderate disability (WHODAS 2.0 score > 35%), health-related quality of life (measured by EuroQol Dutch EQ-5D-5L) and patient health status (measured by Short-Form Health Survey; SF-12, Dutch, version 2.0) at one and three months postoperatively, intensive care unit admission, length of stay in the intensive-care unit and in the hospital, 90-day mortality, and incidence of postoperative complications (Supplementary Appendix 2).

### 2.6 Study schedule and randomization

Table 1 shows the planned study schedule. As can be seen in the table, all participating wards started in the control phase. After a specific number of patients had been enrolled on that ward, the intervention period was started on the respective ward following a stepped-wedge model (Table 1). The stepped-wedge approach was chosen, as it prevents contamination that could arise from a learning effect when using a dual approach (wireless monitoring, whilst simultaneously randomly assigning patients to standard perioperative care in the same ward). In the intervention group, patients received standard care plus remote wireless monitoring. To treat an equal number of patients in the intervention and control group in both hospitals, wards 1 and 4 were assigned to Amsterdam University Center and wards 2 and 3 were assigned to University Medical Center Utrecht.

**Table 1:** Stepped wedge study design.

Ward	1	2	3	4	5	6	7	8	9	10	11	12
1	30		30	30	30	30	30	30	30	30	30	30
2	30	30	30	30		30	30	30	30	30	30	30
3	30	30	30	30	30	30	30		30	30	30	30
4	30	30	30	30	30	30	30	30	30	30		30

Study timeline overview according to study block (twelve blocks, each consisting of 30 patients), study carried out on four wards in a cluster stepped-wedge model: all wards started including control patients first, switched over to intervention periods after inclusion of a predefined number of control patients; light grey: control period; white: transition period; dark grey: intervention period.

The study was intended to be conducted in twelve study blocks: eleven measurement blocks and one transition period (Table 1). The exact duration of each study block was governed by study enrolment. During the transition period, nurses of the respective wards were trained to use the wireless monitoring system. Research nurses and medical students were continuously available for questions and support. During the intervention period, a research nurse or medical student visited the respective ward at least once a day for questions and to motivate protocol adherence. To ensure an equal number of patients across the two study arms, a switch to the subsequent study phase was made after 30 study patients had been recruited in one study block.

### 2.7 Study termination

As a result of the COVID-19 pandemic, the SHEPHERD trial had to be terminated prematurely. During the COVID-19 pandemic, all major surgery was scaled down in both academic hospitals, to free personnel to care for COVID-19 patients in dedicated “COVID-19 wards” and (expanded) ICUs. Two participating wards were used as COVID-19 cohorts instead of surgical wards. This resulted in a significant delay of study participation in both hospitals. After the COVID-19 pandemic, due to a fusion of the Academic Medical Center (AMC) and Vrije Universiteit (VUmc) hospitals, and subsequent re-organization of care, both participating wards moved to VUmc. Installation of the system and training of the ward staff would have resulted in a further inclusion delay, and further disrupting the symmetry of the stepped-wedge ward inclusion pattern. As a result, it was decided to stop this study prematurely.

In the UMC Utrecht, all participating patients were included in one surgical ward, these were all control patients. The second participating ward in UMC Utrecht would have been a trauma ward. Due to the COVID-19 pandemic and local circumstances, this trauma ward had not yet started recruiting patients. Consequently, all included patients were surgical patients recruited on three wards, namely ward one and four in Amsterdam UMC, Location University of Amsterdam (control and intervention) and ward three in UMC Utrecht (control patients only).

### 2.8 Data collection

Local investigators collected patient and clinical data several time-points during the study (Supplementary Appendix 3). Questionnaires were completed pre-operatively (baseline) and at one and three months postoperatively. Coded data were entered into a Good Clinical Practice compliant electronic Case Report Form (eCRF, Castor EDC, Netherlands).

To minimize loss to follow-up, patients were called by the local investigator three days after the questionnaires were supposed to be filled in and returned, and again after seven days if a questionnaire had not yet been returned. When a patient refused further study participation, this specific time point was marked in the system, and no further information was gathered for the respective patient.

### 2.9 Statistical analysis

#### 2.9.1 Sample size calculation

With 4 wards and 11 study blocks for gathering baseline and intervention data, a total of 44 unique ward blocks (4*11) were defined, equally divided over the baseline and intervention periods. A total of 30 evaluable patients were needed per ward per block to detect an absolute decrease in the percentage of new disability at three months follow-up of 10% in favor of wireless monitoring, when the percentage of patients with new disability is 21% in the standard monitoring group, with a power of 0.8 and a two-sided significance level of 0.05. As mentioned, new disability was defined as an absolute increase in WHODAS (pre-operative value versus 3-months postoperatively) of 5% (28). The total number of evaluable patients needed equals 1,320 (44*30) or 660 per treatment group. Patients changing location postoperatively to another ward were considered drop-out.

#### 2.9.2 Statistics

Descriptive statistics were used to present patient characteristics. Binary and categorical variables are presented as a count and percentage (*n* (%)). Numerical data are presented either as a mean with standard deviation (SD) for normally distributed data, or a median with interquartile range (IQR) for non-normally distributed data.

Due to early termination before the calculated sample size could be reached, the outcome parameters are only presented using descriptive statistics. Statistical analyses were performed according to the intention-to-treat principle (ITT).

## 3 Results

**Figure 2:**
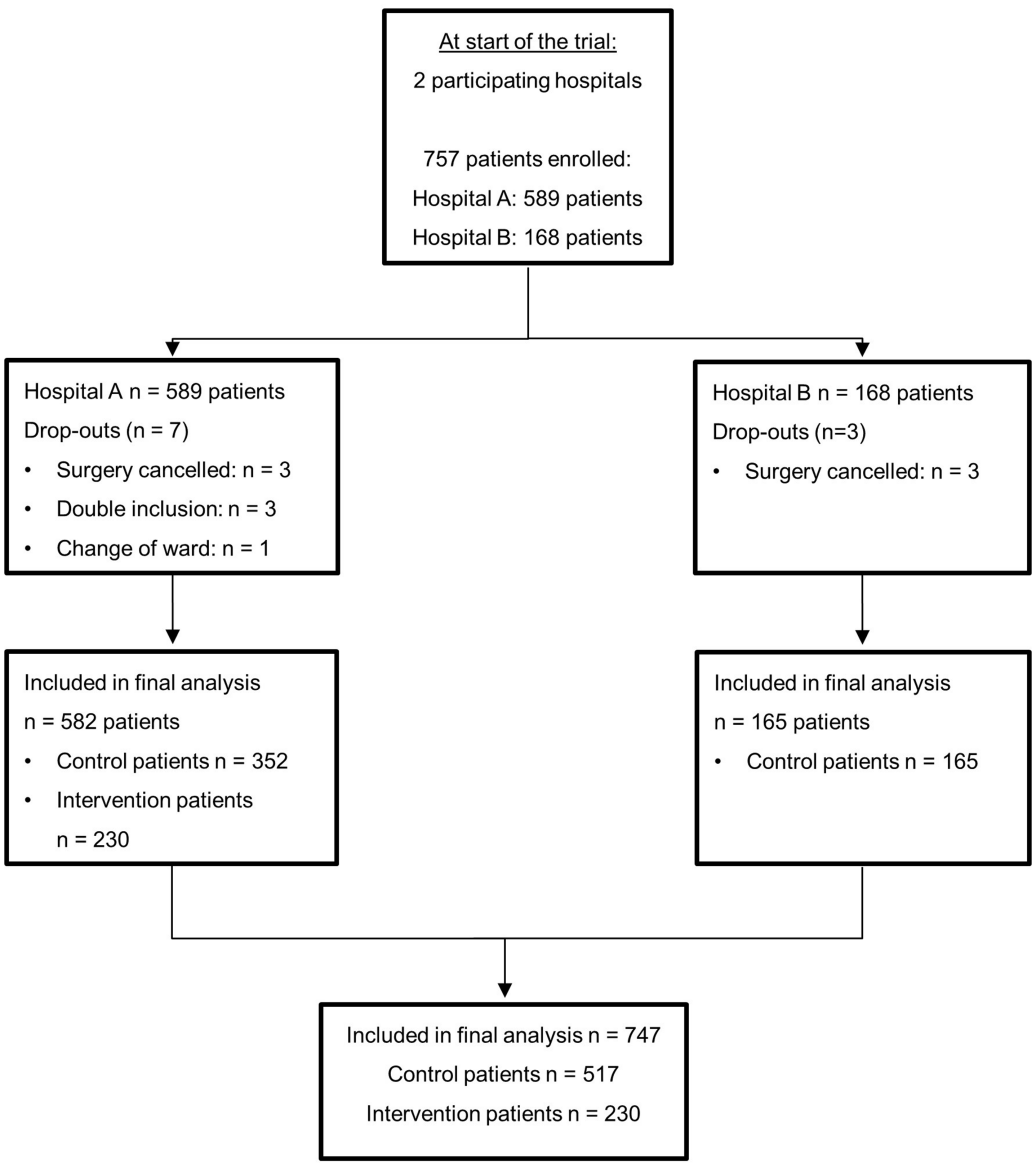
Patient flow diagram. n, number of patients.

Patients were recruited between February 2018 and May 2021. Informed consent was obtained from 757 patients, of which 747 patients were eligible for analysis. In the control group 517 patients participated and in the intervention group 230 patients (Figure 2, Supplementary Appendix 4: study timeline overview).

Baseline patient data are shown in Table 2. Approximately 40% of patients were female, mean BMI was about 26 kg/m^2^ in both groups. Most patients had an ASA physical status of II or III. Regarding comorbid disorders, cancer was present in 58.4% of patients in the control group versus 70.0% patients in the intervention group. Diabetes Mellitus was observed in 16.1% of patients in the control group, versus 11.7% patients in the intervention group. In the control group, 54.4% of patients underwent major surgery, compared to 52.6% of patients in the intervention group.

**Table 2:** Baseline characteristics.

	Control (*n* = 517)	Intervention (*n* = 230)
Mean age, years (SD)	60.1 (13.1)	58.8 (14.6)
Female, *n* (%)	208 (40.2)	98 (42.6)
Mean BMI, kg/m^2^ (SD)	26.2 (4.3)	25.7 (4.7)
Activity level < 4 METs, *n* (%)	19 (3.7)	5 (2.2)
*ASA classification, n (%)*		
I	47 (9.1)	22 (9.6)
II	312 (60.3)	151 (65.7)
III	155 (30.0)	55 (23.9)
IV	3 (0.6)	2 (0.9)
Smoker	64 (12.4)	33 (14.3)
*Comorbid disorders, n (%)*		
Cardiovascular disease	216 (41.8)	89 (38.7)
Stroke/TIA	30 (5.8)	14 (6.1)
Cancer	302 (58.4)	154 (70.0)
Diabetes mellitus	83 (16.1)	27 (11.7)
COPD/Asthma	56 (10.8)	24 (10.4)
Renal disease	35 (6.8)	11 (4.8)
*Severity of surgery, n (%)*		
Minor	0 (0)	1 (0.4)
Intermediate	236 (45.6)	108 (47.0)
Major	281 (54.4)	121 (52.6)

SD, standard deviation; COPD, chronic obstructive pulmonary disease; *n*, number of patients; %, percentage; TIA, Transient Ischemic Attack; MET’s, metabolic equivalents; ASA, American Society of Anaesthesiologists Phys.

In total, 14 patients died within 90 days after surgery, 11 (2.2%) patients in the control group versus 3 (1.3%) patients in the intervention group.

The primary outcome was reported for 604 (80.9%) patients (18 informed consent withdrawals, 139 patients lost to follow up); 435 (84.1%) patients in the control group and 169 (73.5%) in the intervention group. A complete case analysis was used to analyze differences in questionnaire results between groups. New disability at three months after surgery occurred in 43.7% in the control group and in 39.1% in the intervention group (Table 3), an absolute difference of 4.6%.

**Table 3:** WHODAS 2.0 scores.

	Total	Control	Intervention
WHODAS 2.0 completed questionnaire, *n* (%)			
Pre-operatively	742 (99.3)	515 (99.6)	227 (98.7)
1 month	595 (79.7)	427 (82.6)	168 (73.0)
3 months	593 (79.4)	426 (82.4)	167 (72.6)
Completed pre-operative and 1 month	592 (79.3)	425 (82.2)	167 (72.6)
Completed pre-operative and 3 months	590 (79.0)	424 (82.0)	166 (72.2)
New disability*, *n* (%)			
1 month	395 (65.8)	290 (67.0)	105 (62.9)
3 months	256 (42.4)	190 (43.7)	66 (39.1)
WHODAS 2.0 score (%), median (IQR)			
Pre-operatively	8.3 (16.7)	8.3 (16.7)	8.3 (16.7)
1 month	22.9 (29.2)	22.9 (29.2)	22.9 (22.9)
3 months	12.5 (18.8)	12.5 (20.8)	10.4 (16.7)
WHODAS 2.0 score > 35%, *n* (%)			
Pre-operatively	73 (9.8)	48 (9.3)	25 (11.0)
1 month	190 (31.9)	142 (33.3)	48 (28.6)
3 months	74 (12.5)	56 (13.1)	18 (10.8)

WHODAS 2.0, range 0–100%; higher scores mean more disability. *New disability: patients that died or patients with a change in WHODAS 2.0 score of ≥ 5% (for instance pre-operative WHODAS 2.0 score 21%, at three months WHODAS 2.0 score 27%; 27%–21% = 6%, 6% > 5% means new disability). WHODAS 2.0 > 35%: at least moderate disability. IQR: interquartile range, 1/3 month(s): one or three months after surgery.

Pre-operatively, the median WHODAS 2.0 score (*patients that died excluded*) was 8.3% in both groups. At three months, the median WHODAS 2.0 score was 12.5% in the control group, compared to 10.4% in the intervention group: thus, the WHODAS 2.0 scores increased (worsened) in 4.2% of patients of the control group versus 2.1% patients of the intervention group. Moderate disability (WHODAS 2.0 score > 35%) was registered in 13.1% (pre-operatively 9.3%) of patients in the control group (increase of 3.8%), compared to 10.8% (pre-operatively 11.0%) in patients of the intervention group (decrease of 0.2%). There was no difference in median EQ-5D scores at three months after surgery. The median SF-12 score was 46.5 for physical health and 47.1 for mental health in the control group and 44.8 and 48.4 for physical and mental health, respectively, in the intervention group at three months after surgery (Table 4).

**Table 4:** EQ-5D-5L and SF-12.

	Total	Control	Intervention
EQ-5D-5L, median index score (IQR/n)			
Pre-operatively	0.89 (0.2/742)	0.90 (0.2/515)	0.88 (0.2/227)
1 month	0.81 (0.2/592)	0.81 (0.2/425)	0.80 (0.2/167)
3 months	0.87 (0.2/590)	0.87 (0.2/423)	0.87 (0.2/167)
SF-12, median (IQR/n)			
Pre-operatively, physical health	49.8 (17.5/740)	51.1 (16.1/514)	50.9 (16.3/226)
1 month, physical health	40.5 (13.0/589)	40.5 (13.5/423)	40.8 (11.6/166)
3 months, physical health	46.2 (14.9/588)	46.5 (14.8/421)	44.8 (15.0/167)
Pre-operatively, mental health	49.0 (13.7/740)	49.1 (13.6/514)	49.7 (13.3/226)
1 month, mental health	43.0 (16.7/589)	43.7 (16.3/423)	42.3 (16.9/166)
3 months, mental health	47.3 (15.9/588)	47.1 (16.2/421)	48.4 (15.1/167)

*n*, number patients that completed questionnaire; IQR, interquartile range; 1/3 month(s), one or three months after surgery.

Most postoperative complications were surgical in nature, followed by infectious complications (Table 5). Based on descriptive statistics, all groups of postoperative complications (infectious-, cardiac-, pulmonary-, thromboembolic and vascular, renal-, neurologic-, other-, and surgical complications) were detected equally in both groups – or more often in the intervention group. We were not able to record the specific post-operative time-point when developed complications were observed first, as this was not accurately documented in the electronic health record.

**Table 5:** Postoperative complications.

	Total, *n* (%) (*n* = 747)	Control, *n* (%) (*n* = 517)	Intervention, *n* (%) (*n* = 230)
Infectious compl.*	106 (14.1)	69 (13.3)	37 (16.1)
SSI^*^, any	30 (4.0)	20 (3.9)	10 (4.3)
Superficial surgical site	3 (0.4)	2 (0.6)	1 (0.4)
Deep surgical site	9 (1.2)	7 (1.4)	2 (0.9)
Organ space SSI	25 (3.3)	18 (3.5)	7 (3.0)
Pneumonia	55 (7.4)	40 (7.7)	15 (9.1)
Urinary tract infection	17 (2.3)	6 (1.2)	11 (4.8)
Sepsis, Septic shock	24 (3.2)	15 (2.9)	9 (5.5)
Cardiac compl., any*	47 (6.3)	31 (6.0)	16 (7.0)
Myocardial infarction	2 (0.3)	2 (0.4)	0 (0.0)
Cardiac arrest	3 (0.4)	3 (0.6)	0 (0.0)
Heart failure	4 (0.5)	3 (0.6)	1 (0.4)
Arrhythmia	42 (5.6)	27 (5.2)	15 (9.1)
Pulmonary compl., any*	43 (5.8)	26 (5.0)	17 (7.4)
On ventilator 48 h	12 (1.6)	7 (1.4)	5 (2.2)
Unplanned re-intubation	17 (2.3)	13 (2.5)	4 (1.7)
Oedema, fluid overload	26 (3.5)	15 (2.9)	11 (4.8)
Thromb. & vasc. Compl., any	12 (1.6)	7 (1.4)	5 (2.2)
Deep venous thrombosis	8 (1.1)	4 (0.8)	4 (1.7)
Pulmonary embolism	4 (0.5)	3 (0.6)	1 (0.4)
Renal compl., any	21 (2.8)	13 (2.5)	8 (3.5)
Acute renal failure	13 (1.7)	8 (1.5)	5 (2.2)
Progressive renal insufficiency	8 (1.1)	5 (1.0)	3 (1.3)
Neurological compl., any	23 (3.1)	14 (2.7)	9 (3.9)
Stroke, CVA	1 (0.1)	1 (0.2)	0 (0.0)
Delirium	22 (2.9)	13 (2.5)	9 (3.9)
Surgical compl., any*	124 (16.6)	80 (15.5)	44 (19.1)
Anastomotic leakage	102 (13.7)	63 (12.2)	39 (17.0)
Postoperative bleed	21 (3.2)	17 (3.3)	4 (1.7)
Reoperation	26 (3.5)	16 (4.5)	10 (4.3)
Compartment syndrome	0 (0.0)	0 (0.0)	0 (0.0)
Other compl., any*	188 (25.2)	119 (23.0)	69 (30.0)
Allergic reaction	6 (0.8)	4 (0.8)	2 (0.9)
Ileus	28 (3.7)	16 (3.1)	12 (5.2)
Other	175 (23.4)	110 (21.3)	65 (28.3)

*N*, number of patients; %, percentage; SSI, Surgical Site Infection; Compl., complications; Thromb. & vasc. Compl., thromboembolic and vascular complications. *Some patients developed more than one complication.

Median length of hospital stay was 5.9 days (*Interquartile Range* (IQR): 6.4 days) in the control group and 6.0 days (IQR: 8.0 days) in the intervention group. Twenty-two patients (4.3%) in the control group and 13 patients (5.7%) in the intervention group were admitted to the ICU after an initial postoperative stay on the ward. The median length of stay in the ICU after an initial ward stay was 3.4 days (IQR: 11.0 days) and 6.0 days (IQR: 8.6 days) in the control and intervention group, respectively.

## 4 Discussion

### 4.1 Main results

Although this study was terminated after the inclusion of 747 patients, this is still the largest randomized controlled trial investigating continuous wireless monitoring in postoperative patients. In this multicenter stepped-wedge trial, slightly less than halve of the intended absolute reduction of 10% in new disability at three months was observed, namely a reduction of 4.6% in new disability in favor of the patients receiving continuous wireless monitoring on top of standard care as compared to the control group receiving standard monitoring of vital signs.

### 4.2 Study interpretation

Continuous wireless monitoring is a promising technique to reduce *failure to detect* complications and improve patient outcome (29, 30). So far, most continuous wireless monitoring studies have been feasibility-testing studies or clinical validation studies (31–37). A systematic review by Downey et al. (38) suggested clinical benefit of continuous wireless monitoring, however, large randomized controlled trials were still missing (39). Previous studies regarding this topic were retrospective (15, 16), used a before-after design (11, 14, 40) or evaluated only small sample sizes of surgical patient groups (12, 41, 42). Since regretfully in the current multicenter randomized study the intended sample size was not reached, only descriptive statistics were used and therefore any observed difference between control and intervention group study results should be interpreted with caution.

Based on these descriptive statistics, a higher percentage of patients in the control group reported new disability at three months after surgery, and also the mortality rate was higher in the control group compared to the intervention group receiving continuous wireless monitoring. Strikingly, in the control group the percentage of patients with a WHODAS score above 35% percent increased, reflecting at least moderate disability. This increase in WHODAS score above 35% was not observed in the intervention group. The reported WHODAS scores are in line with the primary outcome. These observations might indicate that continuous wireless monitoring in postoperative patients resulted in less (new) disability. However, as mentioned above, study results should be interpreted with caution since statistical analysis was not performed.

Some explanations of the study results seem feasible. First, although the results might indicate that continuous wireless monitoring resulted in less (new) disability and mortality, differences between study groups might be too small to find a significant difference in patient outcome between study groups. Since the study was terminated prematurely, statistical analysis was not performed, and definitive conclusions cannot be made.

An actual lack of sufficient effect could be explained due to malfunction of the continuous wireless monitoring system or response to notifications. However, that seems unlikely in this trial because there was an extensive implementation phase (43) of the continuous wireless monitoring system on the ward and continuous support by medical students. Previous studies demonstrated that the continuous wireless system used in this trial works accurately (22, 25), continuously (24), alarms are generated in time (44) and the system can decrease time to detect patient deterioration (4). Malfunctioning of the continuous wireless monitoring system as explanation for our study results therefore seems unlikely.

Another explanation would be that differences between study groups actually exist as a result of continuous monitoring, as could be explained as follows: postoperative complications are associated with preoperative patient morbidity and will develop after surgery (45, 46). An explanation for the possible increased number of postoperative complications in the intervention group could be that clinicians were more aware of physiological decline due to continuous vital signs monitoring (4, 8, 47) which could have resulted in diagnosing more postoperative complications (48, 49) and ICU admissions for adequate treatment (50). Vital signs of the control group were only infrequently checked manually, and therefore not all deranged vital signs and complications might have been noticed in the control group (5, 6, 51–53). This could have resulted in failure to detect and failure to treat postoperative complications, together leading to failure to rescue, and as a consequence increased new disability and higher mortality rates. Failure to rescue might have occurred more often in the control group (45) and could explain the difference in postoperative complications rates, ICU admissions, WHODAS-data and mortality.

This explanation is attributable to the results of other recent continuous wireless monitoring studies in postoperative patients as well (11, 12, 14–16, 41). In line with our results, these studies in postoperative patients also found a positive effect of continuous monitoring on primary outcomes, however, these trials reported an improvement in patient outcome in different study endpoints. For instance, a decrease in RRT activation was observed in the study of Weller et al. (14) and a decrease in ICU and hospital stay in the study of Brown et al. (15). Continuous wireless monitoring of postoperative patients might have resulted in early recognition of patient deterioration in all studies, however, caregivers response on early recognition might differ and could have resulted in outcome differences between studies (50).

Based on our study results and in line with previous continuous wireless monitoring studies in postoperative patients it seems that continuous wireless monitoring in postoperative patients might be able to reduces failure to rescue. However, as mentioned before our study results should be interpreted with caution.

### 4.3 Strengths

While many trials used postoperative complications or length of stay as primary outcome, in this study new disability was specifically chosen. Improved patient outcome can be measured in different ways and perceptions of “success” of an operation differs widely between doctors and patients. For instance, the exact amount of blood loss during surgery is a meaningful outcome for a surgeon but might not have any meaningful impact on patient health status and thus be less relevant to the patient. In contrast, postoperative complications that result in new disability are highly relevant to the patient (28, 54).

### 4.4 Limitations

Regretfully, as mentioned, the study had to be terminated after the inclusion of 747 patients instead of the expected 1,320 patients. It would be possible to perform a statistical test on these 747 patients, for instance a two-sided Pearson’s chi-square test, with the following hypothesis: Null hypothesis H_0_: π _experimental group (Sensium patch)_ = π _control group_ Alternative hypothesis H_1_: π _experimental group_ ≠ π _control group_. However, given that our *a-priori* sample size calculation is correct, which seems from the preliminary analysis of the data, additional statistical testing reveals that the sample size is too small to find a clinically meaningful significant difference but also does not allow to conclude a lack of effect of the intervention. Together with our statisticians we therefore decided not to do additional testing as we do not want to give the impression that we are able, based on our sample size, to provide solid results. Consequently we only provide descriptive statistics and leave interpretation of the data to the reader. In this discussion we present our own interpretation, which of course are limited to the presented data. These descriptive study results might give rise to extra debate and discussion of remote monitoring and hopefully motivates to perform new studies regarding remote monitoring. As we mentioned several times, all study results have to be interpreted with caution and study results remain inconclusive and need further investigations.

Because the calculated sample size was not reached, there was a difference in group size between control-and intervention group and a difference in baseline characteristics. The extent to which these differences contributed to study outcomes is unknown. Furthermore, all monitored patients came from one department that randomized first to step over to the intervention phase. Only one ward stepped over to the intervention phase, and differences of care delivered or case mix of patients on this respective ward might have influenced the results. However, the two wards in hospital A have the same case mix and were almost identical regarding to workflow and standards of care. Some nurses worked on both wards. There might have been a difference between hospital A and B regarding the control patients (no intervention phase in hospital B), however, both institutions are academic, government owned hospitals in an urban setting, 40 kilometers apart, using the same international guidelines (ERAS guidelines). It seems therefore unlikely that differences in wards have significantly influenced the observed results. It is, however, conceivable that not continuous wireless monitoring itself but the training that nurses received (55) or the Hawthorne effect (56) contributed to the observed differences.

A disadvantage of the WHODAS questionnaire to measure new disability is the relative effort it takes to obtain complete questionnaires three months after surgery. Many questionnaires were sent by physical post since many elderly patients did not respond to e-mail. Research nurses needed to call patients to remind them to complete the questionnaires. Those questionnaires mailed to the hospital were entered into the electronic database, but due to missing answers or illegible handwriting, the data were eventually inaccurate. Furthermore, not all patients were reached for follow up, resulting in a missing primary outcome parameter for a substantial number of patients. We defined a new disability as a change of 5% or more in the WHODAS score. It was previously suggested that patients with a score < 16% have an acceptable symptom state and can be considered disability free (28). Both of our groups had scores less than 16%, namely 12.5% and 10.4% in the control group and intervention group, respectively. Based on this one could argue that both groups were disability free, showing that our patient population was probably not at very high risk. However, we namely looked for a change in disability, and found the observed results presented.

## 5 Conclusion

This study is the largest randomized controlled trial in surgical patients assessing the effect of continuous wireless monitoring on postoperative outcome after intermediate and major surgery. While it seemed that patients in the intervention group receiving continuous wireless monitoring experienced less new disability than patients in the control group, study results have to be re-confirmed in future adequately powered out-come studies.

## Data availability statement

The raw data supporting the conclusions of this article will be made available by the authors, without undue reservation.

## Ethics statement

The studies involving humans were approved by medical ethics committee of Amsterdam University Medical Centre. The studies were conducted in accordance with the local legislation and institutional requirements. The participants provided their written informed consent to participate in this study.

## Author contributions

LP: Conceptualization, Data curation, Formal analysis, Investigation, Methodology, Project administration, Software, Supervision, Validation, Visualization, Writing – original draft. MB: Data curation, Investigation, Project administration, Supervision, Writing – review & editing. PL: Conceptualization, Funding acquisition, Resources, Supervision, Writing – review & editing. EN: Data curation, Investigation, Resources, Supervision, Writing – review & editing. MV: Data curation, Investigation, Project administration, Software, Writing – review & editing. JB: Conceptualization, Data curation, Investigation, Methodology, Project administration, Resources, Software, Supervision, Validation, Writing – review & editing. CW: Data curation, Project administration, Writing – review & editing. JS: Data curation, Formal Analysis, Investigation, Methodology Resources, Software Writing – review & editing. LV: Data curation, Investigation, Project administration, Writing – review & editing. MR: Data curation, Investigation, Project administration, Software, Supervision, Writing – review & editing. JR: Conceptualization, Resources, Supervision, Writing – review & editing. MD: Conceptualization, Resources, Supervision, Writing – review & editing. MH: Conceptualization, Funding acquisition, Investigation, Methodology, Resources, Supervision, Validation, Writing – review & editing. CK: Conceptualization, Formal Analysis, Funding acquisition, Investigation, Methodology, Resources, Supervision, Writing – review & editing. BP: Conceptualization, Data curation, Funding acquisition, Investigation, Methodology, Project administration, Resources, Supervision, Validation, Writing – original draft.
