# Supplementary material for: Surveillance of high-risk early postsurgical patients for real-time detection of complications using wireless monitoring (SHEPHERD study): results of a randomized multicenter stepped wedge cluster trial
Source: Front Med (Lausanne). 2024 Jan 5;10:1295499. doi: 10.3389/fmed.2023.1295499 (PMC10796990; doi:10.3389/fmed.2023.1295499)
Supplement: Supplementary file 1 [file Data_Sheet_1.docx]

Supplementary Material

**Appendix A**

Table A1: Modified Early Warning Score[1] and Escalation protocol

**Modified Early Warning Score[1]**

| Score | 3 | 2 | 1 | 0 | 1 | 2 | 3 |
| --- | --- | --- | --- | --- | --- | --- | --- |
| Respiratory Rate (breath/min) |  | <9 |  | 9-14 | 15-20 | 21-30 | >30 |
| Oxygen saturation (%) | <90 |  |  |  |  |  |  |
| Heart frequency (rate/min) |  | <40 | 40-50 | 51-100 | 101-110 | 110-130 | >130 |
| Systolic blood pressure (mmHg) | <70 | 70-80 | 81-100 | 101-200 |  | >200 |  |
| Urine production | <75 ml in last 4 hours: 1 point | | | | | | |
| Consciousness |  |  |  | *A | V | P | U |
| Temperature (°C) |  | <35.1 | 35.1-36.5 | 36.6-37.5 | >37.5 |  |  |

*AVPU: alert/verbal/painful/unresponsive

**Escalation protocol:**

1. MEWS ≥ 3: call physician
2. Physician formulates diagnostic/treatment plan within 30 minutes
3. Evaluation treatment plan within 60 minutes
4. No patient improvement: activate rapid response team (RRT)
5. RRT can be activated at any moment if felt adequate by attending nurses

## Appendix B. Definitions of postoperative complications[2 3]

1. *Infectious complication, including:*

- Superficial surgical site (SSI):
  - purulent drainage from superficial incision or deliberate opening of superficial incision by surgeon and clinical signs of local inflammation (e.g. pain, tenderness, swelling, redness)
  - wound disruption
- Deep surgical site (SS): defined as purulent drainage from superficial incision or deliberate opening of superficial incision by surgeon and clinical signs of local inflammation (e.g. pain, tenderness, swelling, redness)
- Organ space SSI: defined as purulent drainage from superficial incision or deliberate opening of superficial incision by surgeon and clinical signs of local inflammation (e.g. pain, tenderness, swelling, redness)
- Pneumonia: defined as purulent sputum or isolation of a pathogen from sputum culture or blood culture and clinical symptoms (dyspnoea, fever, cough) or a consolidation or pleural effusion on chest radiograph
- Urinary tract infection: defined as fever (T ≥ 38.5 degrees Celsius), leucocytosis and a positive urine culture
- Sepsis/Septic shock:
  - sepsis: defined as systemic inflammatory response syndrome (SIRS) in response to a proven or suspected infection
  - septic shock: A subset of sepsis in which particularly profound circulatory, cellular, and metabolic abnormalities are associated with a greater risk of mortality than with sepsis alone.

1. *Cardiac complication, including:*

- Myocardial infarction: To confirm the diagnosis of PMI the following criteria were adopted: elevated troponin greater than ten times the 99^th^ percentile in patients with normal baseline; associating to a new pathological Q wave or new left bundle branch block (LBBB) on ECG or coronary angiography showing occluded graft or native vessel or image test showing loss of viable myocardium or new abnormal segmental movement
- Cardiac arrest: asystole or cardiopulmonary resuscitation because of ventricular fibrillation or ventricular tachycardia
- Heart failure: Clinical symptoms susceptible for heart failure.
- Arrhythmia: ECG confirmed diagnosis of Arrhythmia

1. *Pulmonary complication, including:*

- On ventilator 48h
- Unplanned re-intubation
- Pulmonary oedema: findings of pulmonary oedema in chest x-ray, for example pleural effusions, interstitial oedema, Kerley B lines.

1. *Thromboembolic and vascular complication, including:*

- Deep venous thrombosis
- Pulmonary embolism

1. *Renal complication, including:*

- Acute renal failure: AKI can be diagnosed if any one of the following is present: Increase in SCr by ≥0.3 mg/dl (≥26.5 μmol/l) within 48 hours; or increase in SCr to ≥1.5 times baseline, which has occurred within the prior 7 days; or urine volume < 0.5 ml/kg/h for 6 hours.
- Progressive renal insufficiency: progression of renal function in patients with already established renal insufficiency.

1. *Neurological complication, including:*

- Stroke, CVA: new neurological disability with cerebral haemorrhage or infarction on computed tomography scan or autopsy.
- Delirium: Acute and fluctuating alteration of mental state of reduced awareness and disturbance of attention.

1. *Surgical complication, including:*

- Anastomotic leakage: defined as presence of luminal contents through anastomotic site causing local inflammation or SIRS, or leak detected on imaging studies in combination with clinical signs of SIRS
- Postoperative bleed: defined as abnormal postoperative bleeding needing surgical (i.e. reoperation) or endovascular (i.e. coiling) treatment
- Reoperation
- Compartment syndrome

1. *Other complication, including:*

- Allergic reaction
- Ileus: obstructive of paralytic, confirmed by radiology investigation
- All other: any other complication not listed above, including blood transfusion and compartment syndrome

**Appendix C**

Table B1: Schedule of enrolment, intervention, and assessment of patients

| **Time point** | **T1** | **T2** | **T3** | **T4** | **T5** | **T6** |
| --- | --- | --- | --- | --- | --- | --- |
| *Enrolment:* |  |  |  |  |  |  |
| Eligibility screening | X |  |  |  |  |  |
| Informed consent | X |  |  |  |  |  |
| *Intervention*: |  |  |  |  |  |  |
| Intervention |  |  | X |  |  |  |
| *Assessments:* |  |  |  |  |  |  |
| Baseline patient characteristics | X |  |  |  |  |  |
| Baseline questionnaires | X |  |  |  |  |  |
| Data of surgery and anesthesia |  | X |  |  |  |  |
| Postoperative clinical course and adverse events |  |  | X | X | X | X |
| One-month questionnaires |  |  |  |  | X |  |
| Three-months questionnaires |  |  |  |  |  | X |

T1: Enrolment and baseline assessment (1 day before surgery)

T2: intraoperative assessment

T3: during postoperative hospital stay/ ward admission after trauma

T4: day of discharge assessment

T5: one month postoperatively

T6: three months postoperatively

Appendix D

Table C1: study timeline inclusion overview

| Ward | 1 | 2 | 3 | 4 | 5 | 6 | 7 | 8 | 9 | 10 | 11 | 12 |
| --- | --- | --- | --- | --- | --- | --- | --- | --- | --- | --- | --- | --- |
| 1 | 47 |  | 30 | 30 | 30 | 30 | 30 | 30 | 30 | *20* | 30 | 30 |
| 2 | 30 | 30 | 30 | 30 |  | 30 | 30 | 30 | 30 | 30 | 30 | 30 |
| 3 | 30 | 30 | 30 | 30 | 30 | *15* | 30 |  | 30 | 30 | 30 | 30 |
| 4 | 30 | 30 | 30 | 30 | 30 | 30 | 30 | 30 | 30 | 35 |  | 30 |

Underlined text: patients included and study block fulfilled, italic text: patients included, study block not jet fulfilled, normal text: patients not included.

**References**

1. Stenhouse C, Coates S, Tivey M, et al. Prospective evaluation of a modified Early Warning Score to aid earlier detection of patients developing critical illness on a general surgical ward. Br J Anaesth 2000;**84**(5):663P doi: <https://doi.org/10.1093/bja/84.5.663>.

2. Le Manach Y, Collins G, Rodseth R, et al. Preoperative Score to Predict Postoperative Mortality (POSPOM): Derivation and Validation. Anesthesiology 2016;**124**(3):570-9 doi: 10.1097/aln.0000000000000972

3. TRACE Study Investigators. Routine Postsurgical Anesthesia Visit to Improve 30-Day Morbidity and Mortality: A Multicenter, Stepped-Wedge Cluster Randomized Interventional Study (the TRACE Study). Ann Surg 2021 doi: 10.1097/sla.0000000000004954
